# Thoughts on Ill-Health

**Published:** 1890-08-30

**Authors:** 


### Thoughts on Ill-Health.

### III.—MORAL HEALTH.

We have a few words more for those whose temptation it is to make too much of their ill-health. It is well to remember that there is such a thing as moral, as well as physical, health, and that the one must not be lost sight of in the (too-often vain) pursuit of the other. Ill-health is terribly prone to lead to selfishness, as well as egotism; and it is surely advisable sometimes to subordinate health to other considerations, to run the risk, let us say, of a slight attack of indigestion rather than wound the feelings of a kind hostess, who, with the best intentions in the world, sets you down to a dinner-table over which your eyes rove in vain for something that you may with perfect safety eat. Or, again, you consult a London physician, and are told that a winter in a warmer climate may have a beneficial effect; and you know as you listen to him that if you fall in with his suggestion an aged mother, or a nervous, careworn wife must be left to mourn and fret herself over your absence. What course shall you pursue? Your friends will not take upon themselves the responsibility of pressing you to remain at home; and so much depends upon the exact circumstances of the case that no general law could possibly be laid down. But certain it is that in many such cases a man would gain more than he would lose by giving up the search for health. And the gain might not only be moral—it would very probably be physical also. So intimately are the mind and body connected one with another, that the very concentration of thought upon one's ailments tends to give those ailments prominence; and it has been often observed that persons suffering from nervous complaints are never so well as when absorbed in the illnesses or the troubles of others. This does not mean that nervous complaints are imaginary ones—probably they are the most trying, and the most painful, of all; but it does mean that they are apt to become worse if dwelt upon, and that they should never be allowed to fill up the horizon of our thoughts.

"Sweet self-pity," again, should be indulged in but rarely. "I'm anything but what I was; I'm losing flesh rapidly—I am, indeed," says a stout, burly man, the tears rising in his eyes as he speaks. Another waxes eloquent over the agony he endured when a tooth was pulled out, and the strange exhaustion and lassitude from which he suffered for two or three days afterwards—quite forgetting that he would very likely have pooh-poohed the whole affair if a friend—and even a friend in equally poor health—had been in the dentist's chair instead of himself. Well, human nature is human nature; my affairs cannot but be more important to me than anybody else's; and although we smile at hearing from De Quincey that when he entered upon his college course, the great anxiety concerning Napoleon's movements, which was then agitating the public mind, "a little divided with me the else monopolising care attached to the solemn act of launching myself upon the world," we perceive that this was no more than was natural, and that we ourselves should probably have felt just the same. But we all need, and such of us as are in delicate health specially need, to guard ourselves against spending an excessive amount of love, pity, interest, and care upon ourselves; from such excess spring egotists, bores, narrow-minded, self-centred persons, who feel themselves the be-all and end-all of existence.

But if ill-health brings many troubles and dangers with it, let us also remember that it cannot utterly spoil our lives, unless we ourselves will have it so. Much of the best work in the world has been done by men and women who have had to contend against much physical discomfort and pain. Throughout the course of what someone has called "that long disease, his life," Robert Hall preached with eloquence and vigour, even forgetting the agonising pains which racked him, until the sermon was over, and he had once more leisure to suffer. Darwin's biographer, again, tells us that after a certain period in his life, he never passed a day without some hours' great physical discomfort, and that he was often laid aside for weeks together; yet it was after that period that his greatest works were written—a marvellous victory, when one thinks of it, of the vigorous mind over the frail and disordered body. And for those who do not aspire to do any great thing in the world—whose aim, perhaps, is to be rather than to do, surely they may take comfort in the thought that pain and weariness have lessons to teach which perhaps could never be learnt without them; that every experience, however painful, brings with it an added power of sympathising with the troubles of others; that the noble and patient endurance of trials is appreciated in the long run by on-lookers, even though the full extent of those trials may never be fully understood; and that the sweetness and patience which come of willing submission to a hard lot are, though hardly won, gifts which Heaven bestows, and "no man taketh away."

